# Experiences and perceptions of students in occupational therapy regarding the use of desktop virtual environments-based simulation: a qualitative study

**DOI:** 10.1186/s12909-025-07495-y

**Published:** 2025-07-01

**Authors:** Zhizhuo Wang, Peiyun Wu, Shaoyun Shi, Weiwei Zhang, Cheng Lin

**Affiliations:** 1 Department of Rehabilitation Medicine, School of Health, Fujian Medical University, Fuzhou, 350122 China; 2 Department of Rehabilitation Medicine, Fuzhou Second Hospital, Fuzhou, 350007 China

**Keywords:** Virtual environments, Virtual simulation, Healthcare professional education, Students’ perceptions, Qualitative research

## Abstract

**Background:**

Desktop virtual environments are widely used within educational contexts. Despite virtual simulation being well established in occupational therapy practice, this pedagogical strategy remains underutilized in occupational therapy education curricula. Thus, this study aimed to explore how students perceived the desktop VE-based simulation learning to better inform the usefulness of integrating virtual simulation into curricula.

**Methods:**

A qualitative methodology was employed to explore students’ experiences and perceptions of engagement in the desktop-based virtual environments learning process. An interview guide was developed through consultation with faculty members based on Kolb’s experiential learning theory. The interviewees were randomly selected via computer-generated randomization from a pool of 53 invited students studying occupational therapy. Based on the principles proposed by Francis and her colleagues, data saturation was reached by the 18th interview. All interviews were audio-recorded, transcribed verbatim, and analyzed using Braun and Clarke’s thematic analysis framework.

**Results:**

Four key themes were identified: (1) concrete experience: the success of D-VSim rests on resources; (2) reflective observation: resource-abundant D-VSim leads to the success of being complementary to conventional PowerPoint learning; (3) abstract conceptualization: the development in learning through dual visualization and manipulation creates the “visual flow”; (4) action experimentation: the D-VSim provides multi-sensory immersive opportunities for the “repeated” activity. These four key themes were illustrated in the ascending spiral with each layer representing one theme parallel to its corresponding sub-themes.

**Conclusion:**

This study supported that structured reflection enhanced learning through the medium of virtual simulation, which promoted the desktop VE-based simulation in other areas of healthcare professional education.

## Background

Virtual environments (VE) are defined as the computer-generated, three-dimensional environments in tandem with activities of their users synchronously. Specialized input and display devices are used in combination to transmit and receive data in real time. Desktop-based virtual environments are also widely used as they can be run through standard computer monitors and input devices (e.g., keyboard, mouse, touchscreen or joystick) [1]. Even though virtual environments can produce valuable immersive experiences, there are several reasons why the desktop-based virtual environments are preferable to be used within educational contexts [1]. Desktop-based virtual environments can facilitate peer and tutor interaction as well as being relatively affordable for most higher education institutions. Furthermore, social distancing was necessary in the period of COVID-19, which challenged the traditional contact learning [2]. Therefore, desktop-based virtual environments were considered as a promising platform to improve medical education without jeopardizing students or teachers. A rapid review also reported that the best way to address the pandemic was to utilize the resources available today [3].

Simulation is the re-creation, either partially or totally, of a real-life scenario [4]. The optimal level of realism depends on the learning goals—sometimes simpler is better [5]. A number of reviews and meta-analyses have found that simulation, when employed as a supplementary teaching tool, can significantly promote knowledge acquisition, develop clinical/practical skills, and facilitate self-confidence and learning satisfaction [6–10]. Virtual simulation, defined as a partially immersive, screen-based experience, has successfully provided education and assessment activities throughout a variety of healthcare disciplines [11]. Healthcare professional students appreciate the opportunity that simulation provides to systematically apply their theoretical knowledge to tackle real-world problems in a secure and realistic milieu [12]. Evidence also demonstrates that virtual simulation can be used to aid healthcare providers in the pre-clinical education to develop cognitive skills to enhance person centered care and contribute to evidence-based practice [13]. To be qualified as a competent entry-level practitioner, healthcare professional students need to develop clinical and communication skills [14]. Desktop-based virtual environments, as the virtual simulation tool, present simulated environments where users can interact through computer hardware and software without physically being in real contexts. Thus, it is valuable to explore students’ perceptions of desktop-based virtual environments in developing their skills in healthcare professional education.

Healthcare professionals encompass a variety of health-related specialties, such as occupational therapy [15, 16]. Although studies have attempted to determine the effects of virtual simulation in healthcare professional education, such as nursing and dentistry education [17, 18], studies focusing on occupational therapy education are limited [19, 20]. Hence, the aim of the study was to investigate occupational therapy students’ perceptions of engagement in desktop-based virtual environments, in order to integrate or embed desktop-based virtual environments in healthcare professional education for supplementing conventional teaching and learning as well as providing versatile opportunities for students to learn in the digital world.

## Methods

### Participants

A qualitative methodology was employed to provide perspectives of engagement in the desktop-based virtual environments learning process. The study adhered to the Consolidated Criteria for Reporting Qualitative Research (COREQ), a 32-item checklist designed to ensure methodological transparency, minimize bias, and enhance the credibility of qualitative findings through systematic reporting [21]. A total of 53 students studying occupational therapy were purposively invited to participate in the desktop-based virtual environments learning study. The invited students were contacted via email with detailed information on the study attached. The interviewees were randomly selected via computer-generated randomization from a pool of 53 invited students studying occupational therapy. Based on the principles proposed by Francis and her colleagues [22], the initial analysis sample was set as 10, and 20 shared codes (codes mentioned by at least two participants) were yielded after 10 interviews. The following three interviews (P11, P12, P13) continued to generate additional four shared codes until at the 18th interview by applying the stopping criterion (i.e., the following three successive interviews with no new shared codes). Finally, data saturation was reached by the 18th interview.

### Procedures

Kolb’s experiential learning theory [23], a continuous process whereby knowledge is created via transformation of experience, was used as the a priori framework in this study to fit well with the experiences and perceptions of included students. Experiential learning focuses on grasping experience and transforming it into new means of thinking and behaviors [24], consisting of four phases: (i) concrete experience where the student is involved, (ii) reflection on the concrete experience, (iii) abstract conceptualization where the student contemplates thoughts and reflections to recognize the significance of the concrete experience and improve the consequence by doing differently next time, and (iv) active experimentation using what was learned to orchestrate future learning [25]. A large number of college students born from 1995 onward (Generation Z) are commonly engaged with the virtual world and tend to use their own digital environments that certainly influence their learning and understanding. These generations tend to be “reflection light”– like to access information and get tasks completed quickly [26]. Kolb’s experiential learning theory, which has already been employed as a theoretical framework in the context of virtual or digital simulation in health sciences [27, 28], was adopted as the a priori framework in this study to gain insights into learning.

**Fig. 1:**
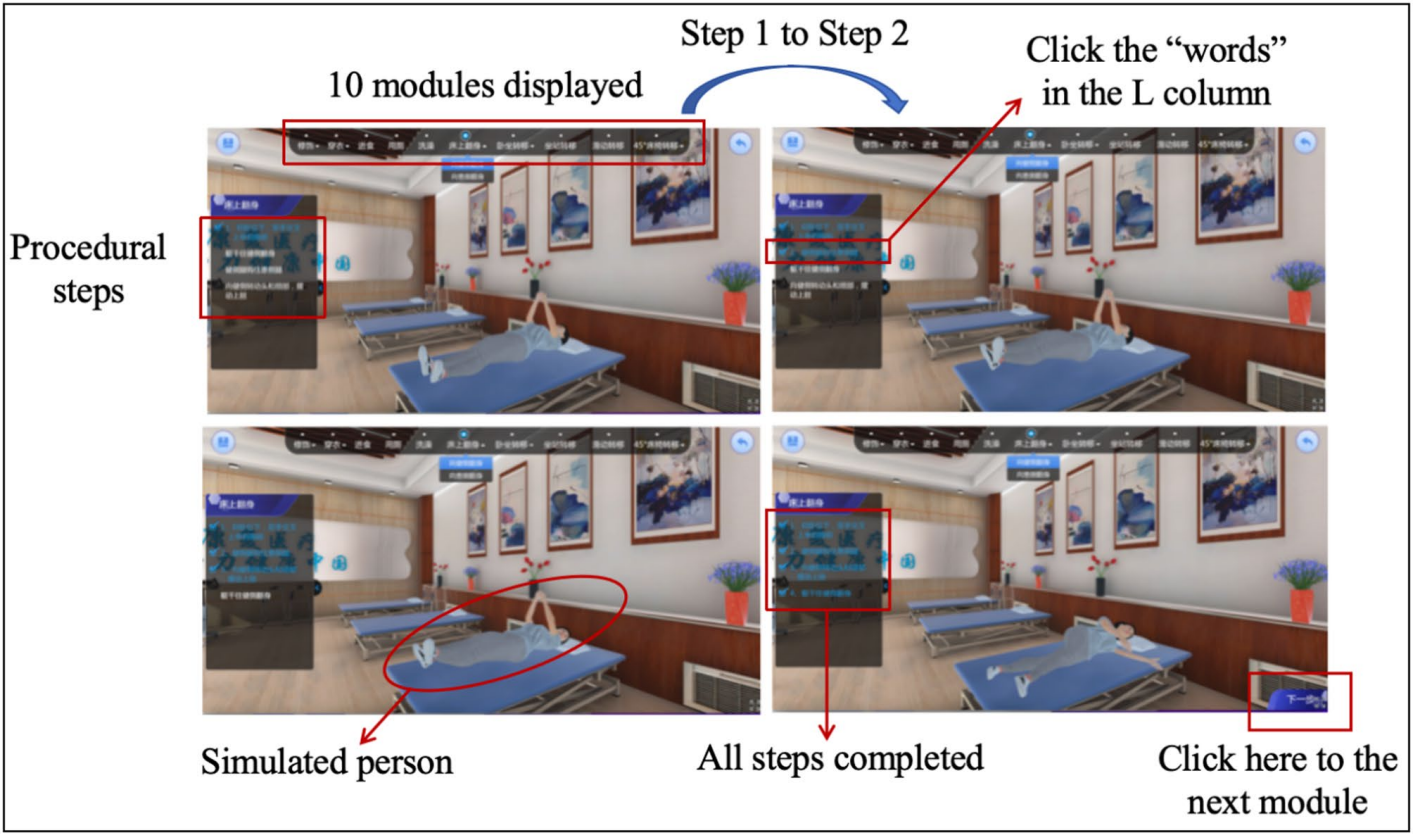
Procedural example of the “Bed mobility” module of the D-VSim ADL training packet

This study was reviewed by the Fujian Medical University Biomedical Research Ethics Review Committee and officially granted exemption (2022 Fuyi Ethics Review No. 169). Verbal informed consents were obtained from all participants prior to commencement of the study. The included students were required to participate in desktop VE-based simulation learning in the experiential laboratory section of the occupational therapy course [section: Activity of Daily Living (ADL) Training]. Students were provided with a user ID and password to access the desktop VE-based simulation learning platform (hereafter called “D-VSim”). The D-VSim was developed by Fujian Medical University and was applied in the occupational therapy experiential laboratory course to healthcare professional students studying occupational therapy, which was accessed through the link: https://adlvr.fjmu.edu.cn/exp/index.html?undefined. The D-VSim ADL training packet included the following 10 modules: “Grooming”, “Dressing”, “Feeding”, “Toilet use”, “Bathing”, “Bed mobility”, “Transfer from lying to sitting position”, “Transfer from sitting to standing position”, “Sliding transfer”, and “45° transfer from bed to chair”. The scenario in each module was simulated as realistic as the Chinese clinical context in order to prepare students for future placement. Taking the “Bed mobility” module as an example, students were required to use the indications provided in the left column to instruct the simulated patient with right hemiplegia to achieve bed mobility from the supine to left-side (or healthy-side) lying position, as shown in Fig. 1. After completing the tasks of the “Bed mobility” module, students were required to answer several online quizzes based on the information provided and knowledge learned. When students finished all 10 modules as required, they were provided with a report where their performance in each module would be shown. As the students were unfamiliar with the D-VSim, they were given approximately 10 min teacher-guided orientation prior to immersion in the ADL training packet. Following teacher-guided orientation, students were required to practice the 10 modules of the ADL training packet.

### Data collection

The interview guide was developed through iterative consultations with two occupational therapy faculty members to ensure content validity. The designed questions were open-ended and intended to facilitate the participants’ responses as much as possible. The development of the topic guide was based on Kolb’s experiential learning theory (i.e., the theoretical framework used in this study). A set of questions of the topic areas were formulated in correspondence to each of four phases of the experiential learning: concrete experience, reflective observation, abstract conceptualization, and active experimentation. The topic areas aligned with experiential learning were as follows: (1) the experience of healthcare professional students when engaging in the desktop VE-based simulation; (2) the facilitators of using the desktop VE-based simulation; (3) the barriers encountered in this process. The one-on-one semi-structured interviews were conducted in Mandarin in April 2023. Each interview ranged from 30 to 60 min in length. All interviews were audio-recorded and subsequently transcribed verbatim. The transcripts were returned to the participants for clarification and confirmation.

### Data analysis

**Fig. 2:**
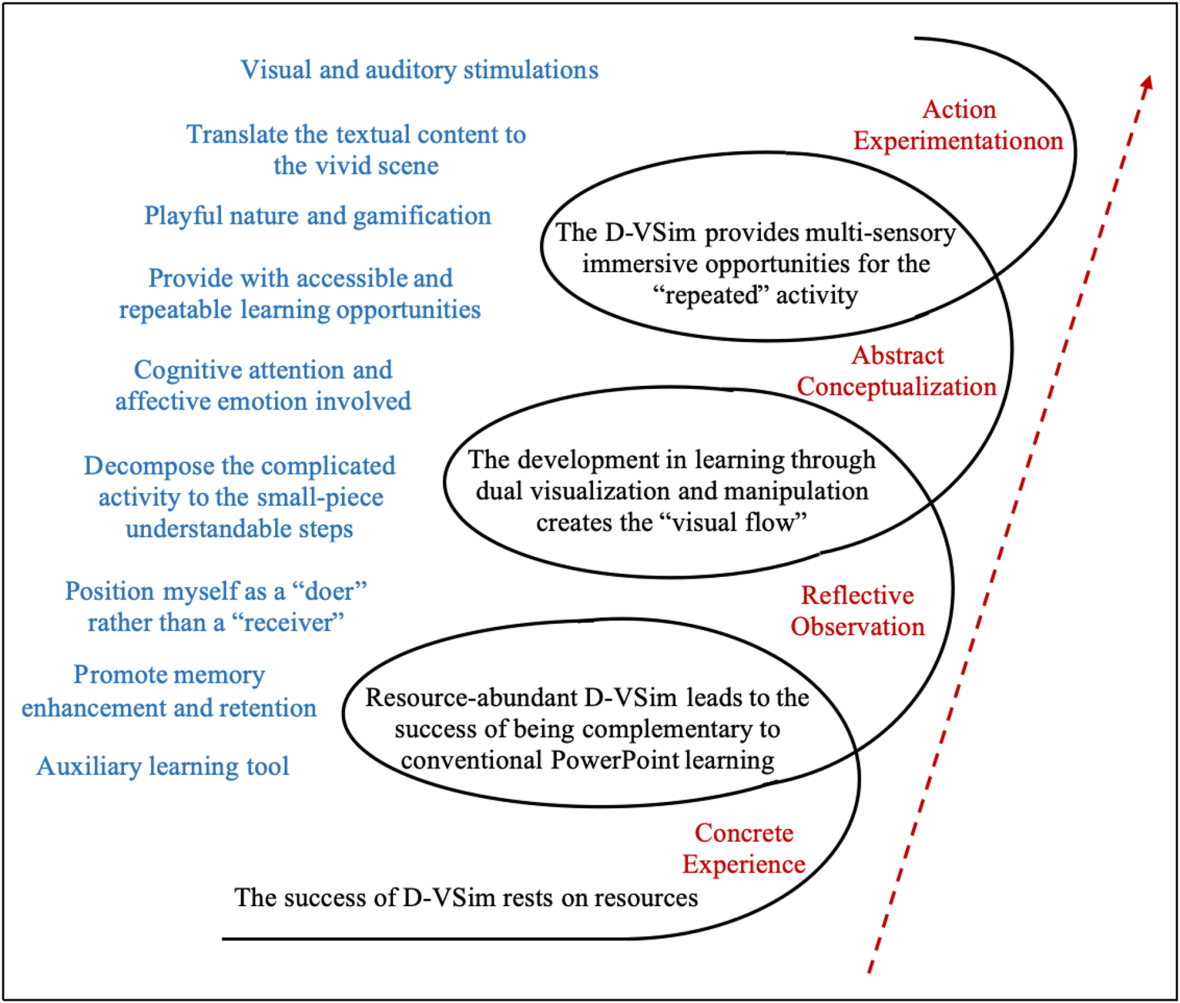
The framework of the themes and sub-themes

Data analysis was performed in accordance with the procedure of thematic analysis proposed by Braun and Clarke [29]. Thematic analysis is defined as a qualitative descriptive method to identify, analyze, and report patterns (themes) within textual data [30]. The full text was read repeatedly by two researchers (ZW and PW) for data familiarization. Several meetings were held to discuss the overall understanding to reach a consensus regarding the essential meaning of the data with initial coding produced manually. Initial codes were then condensed to identify themes. Other research team members (SS and CL) then reviewed the themes to fully reach a consensus across identified themes to inform Fig. 2. The process of data analysis moderation was essential to mitigate potential biases.

Trustworthiness of this study was supported by employing Lincoln and Guba’s criteria of credibility, transferability, dependability and confirmability [31]. Credibility was confirmed by the following techniques: (1) prolonged engagement with the participants to build trust, secure rapport and clarify responses; (2) member checking with participants to obtain participants’ reactions about emerging interpretations; (3) peer debriefing with participants to review and explore various aspects of the inquiry. Transferability was supported by a detailed description of the setting, the participants and the experiences and processes observed during the inquiry. Dependability and confirmability were established by the inquiry audit trail including six elements (i.e., the raw data, data reduction and analysis products, process note, materials relating to researchers’ intentions and dispositions, instruments development information, data reconstruction products).

## Results and discussion

Among 18 participants, seven were male accounting for 38.89% and eleven were female representing 61.11% of the total. All participants were third-year undergraduate students who had limited prior exposure to virtual learning environments in their academic studies. As shown in Fig. 2, the thematic analysis yielded four overarching themes according to the four stages of Kolb’s experiential learning cycle, which was represented in the bottom-to-top spiral layers: (1) concrete experience: the success of D-VSim rests on resources; (2) reflective observation: resource-abundant D-VSim leads to the success of being complementary to conventional PowerPoint learning; (3) abstract conceptualization: the development in learning through dual visualization and manipulation creates the “visual flow”; (4) action experimentation: the D-VSim provides multi-sensory immersive opportunities for the “repeated” activity.

### Concrete experience: the success of D-VSim rests on resources

A resource-abundant D-VSim supports a satisfactory experience for students. When asked for feedback on the desktop VE-based simulation learning, some participants responded: “*when I complete a certain module in the ADL training packet*,* the system could provide images*,* videos or animations to demonstrate the procedural steps rather than relying solely on text*” [P15]. However, others also commented that “*some operating procedures should be modified simplifier*,* such as*,* the grooming activity*” [P18].

The richness of the system’s resources impresses me a lot. In the conventional PowerPoint learning, I can only depend on the descriptions or pictures in the book to understand the manipulations of each ADL training. Sometimes, I need to explore the external resources on the Internet for clarification and comprehension. Right now, the D-VSim integrates abundant resources (e.g., pictures, videos, animations) to the system, which makes it easier for me to locate and learn. [P14]

### Reflective observation: resource-abundant D-VSim leads to the success of being complementary to conventional PowerPoint learning

One of the “good sides” shared by participants was “*virtual simulation is a useful pedagogical tool to counterbalance conventional PowerPoint learning*” [P2]. It is widely accepted that traditional PowerPoint learning is primarily based upon text-based content. Even if the advantages of virtual simulation outweigh those of conventional PowerPoint learning, it is not aimed at replacing conventional PowerPoint learning but at better complementing and assisting students with it.

The virtual simulation should be used as an auxiliary learning tool. The conventional PowerPoint learning can build the foundation of knowledge acquisition, while the virtual simulation can cement it for reinforcement. To be precise, virtual simulation is better functional as a post-class rather than an in-class learning strategy. [P12]

Participants also reported that they preferred virtual simulation for learning because of its accessibility and repeatable practice opportunities. As one participant explained, “*it gives me maximal opportunities to learn…I can use the iPad and login into the system anytime and anywhere*,* such as library*,* canteen*,* and café”* [P5].

Once the Internet is connected to my device, such as iPad, I can access to the system. It is much easier to operate and control the system. If I have to handle other businesses, I can stop it and resume when available. [P16]

Participants believed that virtual simulation separated the whole skill training process into its small-piece individual components and made it more understandable and straightforward. Examples demonstrated “*how virtual simulation can simplify rehabilitation process for stroke patients*,* such as completing upper body dressing activities or transitioning from a supine to a side-lying position for patients with hemiplegia*” [P4].

The textual description of skill training is lengthy and obscure, particularly when expressed with a cluster of jargon. Fortunately, I can understand the meaning of each paragraph and follow the steps of each activity by using the D-VSim system. It truly saves a great deal of time and effort. [P9]

The findings indicated that desktop VE-based simulation holds unique advantages compared to other traditional teaching approaches in the field of occupational therapy. While traditional PowerPoint learning (e.g., didactic lectures) remains essential for delivering foundational knowledge given their efficiency in delivering standardized content to large cohorts and their alignment with established assessment systems [32], desktop VE-based simulation offers students numerous opportunities to apply acquired knowledge within simulated practice environments. Furthermore, desktop VE-based simulation provides distinct benefits over traditional clinical placements [33] by enabling controlled exposure to a wide range of clinical cases and facilitating immediate and standardized feedback via programmed response systems. Additionally, desktop VE-based simulation enhances the fidelity and consistency of therapeutic scenarios while reducing the self-consciousness often experienced during peer role-playing exercises [34]. Overall, desktop VE-based simulation is not intended as a substitute for existing teaching approaches, but rather serves as a powerful complementary tool.

### Abstract conceptualization: the development in learning through dual visualization and manipulation creates the “visual flow”

A significant majority (90%) of participants highlighted learning as an integrative process involving cognitive attention and affective emotion. It did not stay on the perceptual layer of learning, but the higher cognitive level. Some participants were surprised that “*the time is passing so fast when I am studying*” [P8], which suggested that they may have achieved a state of flow.

When watching the colorful screen, my eyes shine with excitement and cannot leave for a second. When the system shows or reports ‘congratulations’ or ‘pass’, I cannot help jumping up from the chair to give out the inner exhilaration. [P10]

Some participants said that “*I take the initiative to open the system for learning*,* and this behavior or phenomenon has never happened before indeed*” [P7].

In my previous learning period, I was forced to learn by myself or others. It may be ridiculous or incredible, but it is true. In the desktop VE-based simulation learning, I redefine myself as a “manipulator” to orient in different knowledge fields. [P13]

Memorization is a mysterious process. Sometimes, I feel that my brain is full of many things but knowledge. It is desktop VE-based simulation that transforms the knowledge to reality. After this training, my memory seems to have improved a lot. This may be an allusion, but genuinely raise me up. [P11]

Flow is defined as a subjective psychological state where one entirely indulges in an activity [35]. Students in a flow state may have some common experiences, such as attention focused on a distinct goal, a loss of self-consciousness, timelessness or a distorted sense of time, the activity that is intrinsically rewarding [36]. Although flow is a psychological phenomenon, a number of researchers have committed to connect the indefinite thing to the measurable evidence. de Manzano et al. found that flow proneness in daily life was correlated with dopaminergic transmission in the dorsal striatum using a positron emission tomography (PET) [37]. Similarly, a functional near-infrared spectroscopy (fNIRS) study unveiled a positive association between flow and functional activity of the prefrontal cortex (PFC) including attention, cognition, emotion and reward processing [38]. In this regard, desktop VE-based simulation learning is an evidence-based pedagogical strategy that yields activities in the brain to promote cognitive and emotional effects.

“Learning by doing” was a new concept conceived from interviews with participants, which echoed the findings of a study where learning through computer-based virtual system appeared to be effective and skills learned virtually could be transferable to physical reality [39]. The concept of learning by doing, derived by John Dewey, means that students learn best through a hands-on approach [40]. Students involved in the study expressed that desktop VE-based simulation learning made me a “manipulator” and knowledge seemed to be more impressive by combining with hands-on control in place of solo visual control. This effect was evident in a fMRI study where positive feedback was correlated with activations in certain areas of brain, such as the ventral striatum, midbrain and anterior and posterior cingulate cortex [41].

Realizing how much knowledge has retained in the mind is essential to examine the learning outcomes, which is supported by a body of empirical evidence [42]. In addition, this realization will enhance self-confidence, thereby amplifying the passion for learning. Finally, the positive feedback loop in learning emerges through a self-regulated learning cycle wherein successful learning experiences enhance motivation and cognitive engagement, which in turn improve future learning outcomes [43].

### Action experimentation: the D-VSim provides multi-sensory immersive opportunities for the “repeated” activity

Almost all participants reported that the D-VSim not only provided visual perception, but also stimulated auditory sensation, which was consistent with previous research [44]. They also perceived that the D-VSim made repeated activity more immersive and attractive, which could not be achieved with previous traditional training.

Virtual simulation captures my eyes with virtual animation and the simulated person can speak when I am following some operating instructions. It is really impressive that the voice is produced real time as I do. [P3]

Virtual simulation was perceived to “*translate the textual content to the vivid scene*” [P17]. Furthermore, one participant described it as “*virtual simulation gives me an illusion that I am playing games rather than completing learning tasks*” [P1].

It is much fun using the virtual simulation for learning as it changes the original way of thinking that learning is a tedious process. Like game-playing, this virtual simulation learning can evoke similar feelings. Therefore, I can study for longer periods without feeling like giving up. [P6]

Another commented on how the multi-sensory immersive virtual simulation facilitated their learning and understanding by presenting/restoring the textual characters. This outcome was based on that virtual simulation employed some game-design techniques and solutions with the intention of education rather than entertainment [45, 44]. Growing evidence supported that the use of gamification in medical education could contribute to peer collaboration and engagement, boost students’ earning analytic capacity and clinical decision-making ability, as well as provide ample opportunities for intentional and systematic practice in clinical reasoning [46, 47]. However, it is noteworthy that gamification can also lead to some unintended and undesirable consequences [48]. For example, students may shift their attention to game elements (competitive attitude to cross the finish line like a race), which counteracts their performance and even intrinsic motivation to learn knowledge and/or skills [49]. As such, we need to be attentive to the praxis of virtual simulation in medical education to minimize negative impacts of its playful nature and gamification.

Given that any learning effects gained during the virtual simulation were most likely obtained through students’ engagement and experience before and during the virtual simulation, Kolb’s experiential learning theory was shown to be a plausible model compared with other learning theories (e.g., humanistic theory, transformative learning theory, motivational model, and social theory of learning) [24]. Take, for instance, transformative learning theory is used when individuals experience a disorienting dilemma, such as Covid-19. Afterward, individuals may experience one or more of three processes including cognitive rational, extrarational and social critique [50]. In our study, the desktop VE-based simulation was not set as a disorienting dilemma, so transformative learning theory may not be considered as the guiding framework. However, Kolb’s experiential learning theory based on the theory of constructivism is focused on the grasping and transforming experience of learning [51]. As such, optimal learning is dependent on how students experience each phase of the Kolb’s cycle. Specifically, upon completion of the D-VSim, the participating students reflected on observation and manipulation in virtual environments to get prepared for future clinical practice, engulfed in the virtual simulated experience to slower the process of knowledge absorption, and appreciated the important professional concepts.

As virtual simulation is a rapidly developing pedagogy worldwide, the findings of the study may have some useful implications for both educational practice and research. However, several limitations should be considered when interpreting the results. First, the relatively small sample size constrains the generalizability of the results. Moreover, the study was exclusively conducted at a single institution (i.e., Fujian Medical University in China), which may affect the external validity of the findings. The unique cultural and institutional context of medical education in China, including its distinct curricular frameworks, variations in students’ prior training, and institution-specific technological adoption patterns, may influence both the implementation and outcomes of virtual simulation-based learning. Lastly, the findings regarding desktop VE-based simulation are particularly relevant to the occupational therapy field, complementing the existing literature across the health sciences.

## Conclusion

This study demonstrated that desktop VE-based simulation holds significant promise for enhancing occupational therapy education by providing immersive, flexible, and standardized learning experiences. Grounded in Kolb’s experiential learning theory, the findings reveal that this approach not only overcomes spatial and temporal constraints but also fosters deeper engagement through authentic, scenario-based practice. While students responded positively to its usability and versatility, future research should expand its applications across healthcare disciplines, adapting simulations to domain-specific needs. Meanwhile, although the study provided the qualitative descriptive evidence for the use of desktop VE-based simulation in healthcare professional education, future quantitative study is needed to determine effects of desktop VE-based simulation on learning.

Based on the study’s findings, the specific recommendations for educators who wish to implement similar virtual environments in their teaching are as follows: (1) use VE-based simulations to bridge gaps in traditional clinical training, particularly when live patient access is limited; (2) adopt a blended learning approach by integrating pre-simulation preparatory materials (e.g., readings, case reviews) and structured post-simulation debriefings; (3) design simulations that closely replicate real-world clinical settings, incorporating interactive elements such as responsive patient avatars and dynamic, adaptive scenarios to maximize engagement and authenticity; (4) expand student exposure by incorporating multi-therapeutic contexts, such as pediatrics, geriatrics, mental health; (5) collaborate with practicing healthcare professionals to align simulation content with current clinical best practices; (6) structure simulations with tiered difficulty levels (beginner to advanced) to scaffold student learning; (7) continuously refine simulations by gathering and incorporating feedback from both students and instructors.

## Data Availability

The datasets that support the findings are available from the corresponding author or first author upon reasonable request.

## Abbreviations

VE: Virtual environments
COREQ: Consolidated criteria for reporting qualitative research
PET: Positron emission tomography
fNIRS: Functional near-infrared spectroscopy
PFC: Prefrontal cortex
fMRI: Functional magnetic resonance imaging
